# An Uncemented Spreading Stem for the Fixation in the Metaphyseal Femur: A Preliminary Report

**DOI:** 10.1155/2016/7132838

**Published:** 2016-05-15

**Authors:** Daniel Burger, Matthias Pumberger, Bruno Fuchs

**Affiliations:** ^1^Department of Orthopaedic Surgery, Balgrist University Hospital, 8008 Zurich, Switzerland; ^2^Department of Musculoskeletal Surgery, University Hospital Charité of Berlin, 10117 Berlin, Germany

## Abstract

Surgical treatment to restore full range of motion and full weight bearing after extensive femoral bone resection in patients with primary or metastatic femoral tumours is individually challenging. Especially when the remaining distal or proximal bone is very short, a rigid fixation of an implant is difficult to achieve due to the reverse funnel shape of the metaphysis. Herein, we present a novel implant design using a spreading mechanism in the distal part of the prosthesis for rigid, uncemented fixation in the remaining femoral bone after extensive tumour resection of the femur. We present the outcome of 5 female patients who underwent implantation of this spreading stem after extensive proximal or distal femoral bone resection. There was no radiological or clinical loosening or implant-related revision surgery in our follow-up (mean 21.46 months, range 3.5–46 months). This uncemented spreading stem may therefore represent an alternative option for fixation of a prosthetic device in the remaining metaphyseal femur.

## 1. Introduction

Primary bone tumours or metastatic lesions of the femur may represent a remarkable surgical challenge, particularly in reconstructing the full weight bearing function of the lower limb with a good pain relief. With extensive bone resections, treatment options such as allograft-prostheses composites, rotationplasty, and endoprosthetic reconstruction by custom made devices or modular systems are well established.

Although these options offer a wide spectrum of applications, postoperative complications were observed [1, 2], including fractures and aseptic stem loosening of the prosthetic implants. In the literature, the incidence of aseptic loosening after cemented massive prostheses is described within 0–6% [3, 4] and is known as a midterm complication due to anatomical location and dimension of resection as well as the age of the patient [5]. If the remaining bone after resection consists only in the short femoral metaphysis, a rigid fixation of the implant is even more difficult to achieve due to the reversed funnel-shaped anatomy of the metadiaphyseal part of the distal/proximal femur. Unwin et al. [6] postulated the bending moment of the femur, which is related to the offset distance (in the proximal femur higher than in the distal femur) between the long axis of the femur and a line passing from the femoral head to the knee joint, to be another reason for stem loosening. Mechanical analysis showed a decreasing moment from 140 Nm at the level of the greater trochanter to almost zero at the level of the insertion of the anterior cruciate ligament around a dorsiventral axis with increasing moment to a great extent during weight bearing [7] and a transmission of 60% of the applied load on the cemented intramedullary stem to the region of the tip of the stem. Therefore, multiple statements about alternative reconstructions, especially concerning the problem with rigid fixation in short remaining proximally or distally femoral bones, ask for an increasing demand in more innovative modular implants.

Accordingly, we investigated a novel prosthetic design using a spreading mechanism of the distal/proximal part of the prostheses for rigid, uncemented fixation in the remaining bone after extensive tumour resection of the femur.

## 2. Materials and Methods

### 2.1. Patient Population

**Figure 1:**
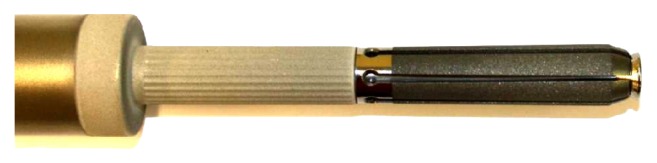
Tip of the spreading stem.

Between November 2010 and December 2011, 5 female patients underwent extensive proximal, diaphyseal, or distal femoral resections and implantation of an uncemented femoral spreading stem (ArgoMedical, Zug, Switzerland) (Figure 1). Three patients had metastasis at diagnosis, one patient presented with a pathological fracture, and one patient had overt systemic metallosis from prior surgeries associated with prosthetic infection. Two of these patients suffered from osteosarcoma and each one suffered from Ewing's sarcoma, undifferentiated sarcoma, and metastatic breast carcinoma, respectively. The average age at the time of surgery was 52 years (range 30 to 82 years). Patients were followed up clinically using the MSTS-Score (musculoskeletal society tumour score) and the TESS-Score (Toronto extremity salvage score) as well as radiological imaging to evaluate rigid fixation postoperatively.

### 2.2. Surgical Technique and Biomechanical Considerations

**Figure 2:**
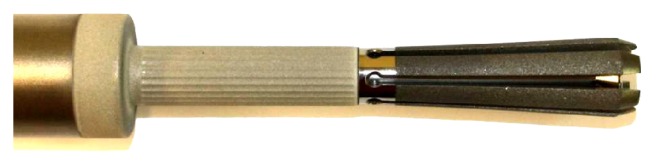
Tip of the spreading stem with open fins.

**Figure 3:**
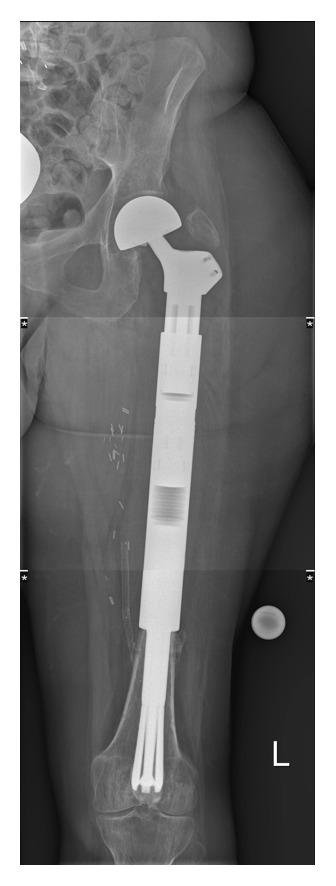
Postoperative X-ray of the implanted spreading stem.

After resection of the tumour-affected part of the femur, the end of the wide part of the area of the noningrowth region of the prosthesis above the ingrowth rigid fluted stem abuts at the remaining distal femur end cylinder in order to achieve the best possible apposition to the shaft after spreading the distal flat fins. The spreading flat hydroxyapatite coated fins (Figure 2) are fashioned by corundum in order to achieve surface enhancement. The nonspreading part of the prostheses, anchoring in the medullary cavity, is constructed by a primary layer of titan covered by a hydroxyapatite layer. Therefore after the osteotomy, the medullary cavity is reamed in a cylindrical shape of 14, 16, or 18 millimetres (mm) of diameter. The depth of the reamed part should be at least 12 centimetres measured from the osteotomy. After double-checking the length of the final prostheses, the stem is adapted into its final position and an Allen wrench is inserted into the hexagon socket of the grub screw placed on the neck of the prostheses. The screw is then turned to achieve a rearward movement of the expanding rod and its guiding bolt. This leads to spreading of the stem with inward movement by the cone shaped tip of the expanding bolt. The six blades of the stem, each of them 70 mm long and hydroxyapatite-coated, are infinitely adjustable. The grub screw has a locking mechanism if the implant is spread to a total angle of 30° on both sides measured from the midline of the prostheses. Biomechanically, a force of 46 kilograms is needed to spread the fins, which is then directly transferred to the surrounding bone. In the surrounding metaphyseal bone, a theoretically maximal spreading force of 2500 kilograms can be generated until the grub screw fails. In practice, fixation strength can be individually adapted and should not be overused because the bone may fail first. Verification of fully spread fins under image intensifier shows adequate opening of the lamellae. Rigid fixation is primarily achieved by deadlock of the spreading fins to the surrounding bone and secondarily by the bony ongrowth to the fins. The intramedullary stem diameter is available in diameters of 14, 16, or 18 mm and has a total length of 120 mm. The prosthetic neckpiece is 150 mm long with an extension module of 65 mm.  Figure 3 shows the X-ray after implantation of an uncemented femoral stem as used in our series.

### 2.3. Postoperative Rehabilitation

 Postoperative rehabilitation was as follows: toe touch weight bearing for the first six weeks followed by gradual increase of weight bearing for the next six weeks and no rotational forces during the entire three months.

## 3. Results

In four out of five cases the TESS- and MSTS-Scores were evaluated between 9.5 and up to 46 months postoperatively. One patient died prior to evaluation of these scores due to metastatic disease. Conventional radiographic evaluation was accomplished from 3.5 months to 46 months (mean 21.46 months) for the longest case. The following table shows the detailed patient data (Table 1). Within this mentioned period of observation, there was no radiological or clinical reported loosening of the implanted femoral spreading stem (five patients with six fixations). Overall, the clinical follow-up of this series is rather small mainly because this implant was used in clinical high-risk situations and the majority of patients in this series have died. The longest surviving patient (number 4 of Table 1) was a 30-year-old female with a pathological fracture through Ewing sarcoma. At the last follow-up, she reported feeling so safe with her implant that she even went skydiving.

## 4. Discussion

With the development of modern chemotherapy, limb salvage surgery in femoral primary or metastatic lesions is well established. Surgical reconstruction remains a challenge when the remaining part of the femur is short, together with the reverse funnel shape and its inherent problems as to biomechanical forces. To anchor the femoral stem in the remaining femoral bone, cement is most often used [8–12]. Several authors describe a high incidence of infection rate and aseptic loosening of the femoral component [8–14]. Farid and Finstein evaluated aseptic loosening with 10% as the most common late complication in their series with cemented endoprostheses of the proximal femur [9, 10]. In our case series, we used a new type of rigid fixation for the femoral stem after extensive resection of the femur without using cement or interlocking pins. This prosthesis uses spreading fins in the shaft to achieve rigid fixation in the residual metaphyseal femoral bone. Due to the high-pressure bone-implant interface attained with the spreading stem, we believe that we present a new possible option for rigid fixation in short remaining femoral bones.

A further possible option for reconstruction after massive femoral resection was created by Johnson in 1994 by its Compress® prostheses [15]. Here, the fixation of the stem in the remaining shaft results from interlocking cross-pins and is supposed to create a rigid, high-pressure bone-implant interface for biologic fixation. In a study by Farfalli et al. [16], however, 12% of the patients (out of 41 with Compress prostheses) had to undergo revision surgery due to fractures and bone resorption within five years using such implants. Interestingly, the revision surgery rate correlated with the stem diameter, which seems to act as a predictor of implant survival [17]. Even though the follow-up of our series is short, we consider the uncemented spreading stem prostheses as an option for thinner shafts where the Compress prostheses is, due to the abovementioned reasons, not recommended.

Limitations of our study include the paucity of biomechanical studies that prove extrastability resulting from the spreading stem and the absence of data describing the stability against rotational forces to the spreading stem. However, related to our preliminary clinical experience, rotatory instability does not seem to be a problem in our follow-up. Further, the overall follow-up in this series is short, and the clinical scores vary. This is mainly explained by the advanced (metastatic) stage of the diseases of the patients included, which affected the functional score more than the rigid fixation of the device in the bone.

Custom-made prostheses for the femur, which are individually designed and manufactured, offer an additional option in reconstruction after extensive resection of the femur. These prostheses are mostly fixed via cortical flanges or interlocking cross-pins [11, 13]. Nevertheless, Natarajan et al. [11] reported a mechanical failure rate of over 13%, even with these individual anatomical shaped prostheses. Another attempt for rigid fixation in ultrashort metaphyseal-condylar segments was described by Cannon et al. [18] with cemented custom-made tumour endoprostheses and cross-stem pin fixation in 32 patients. Their results showed a good reconstructive success with a relatively low complication rate but unfortunately, the company does not produce these custom devices anymore. Disadvantages of these custom-made solutions seem to be the individual manufacturing of these prostheses, which can take some time and delay the treatment of patients and therefore increase the morbidity [19].

## 5. Conclusions

Based on our series, the spreading stem may represent an additional alternative option for fixation of a prosthetic device in the remaining femur after extensive tumour resection. Nevertheless, long-term follow-up of a larger series of patients with this novel implant design is needed to stand the test of time.

## Figures and Tables

**Table 1:** Patient data and results of TESS- and MSTS-Scores (^*∗*^death of patient, n.a. = not available).

Patient	Age at surgery (years)	Tumour	Location	Involved metaphysis	Radiological follow-up (month)	TESS-Score	MSTS-Score (%)
1	61	Breast carcinoma	Proximal femur	Distal	27^*∗*^	24	23.3
2	82	Undifferentiated sarcoma	Diaphyseal femur	Distal and proximal	25^*∗*^	77.3	63.3
3	35	Osteosarcoma	Proximal femur	Distal	3.5^*∗*^	n.a.	n.a.
4	30	Ewing sarcoma	Proximal femur	Distal	46	86.7	78.3
5	52	Osteosarcoma	Distal femur	Proximal	6^*∗*^	90.8	86.7
